# An Innovative Approach for Online Neuroanatomy and Neurorrehabilitation Teaching Based on 3D Virtual Anatomical Models Using Leap Motion Controller During COVID-19 Pandemic

**DOI:** 10.3389/fpsyg.2021.590196

**Published:** 2021-06-28

**Authors:** Esteban Obrero-Gaitán, Francisco A. Nieto-Escamez, Noelia Zagalaz-Anula, Irene Cortés-Pérez

**Affiliations:** ^1^Faculty of Health Sciences, University of Jaén, Jaén, Spain; ^2^Center for Neuropsychological Assessment and Neurorehabilitation (CERNEP), University of Almería, Almeria, Spain; ^3^Department of Psychology, University of Almería, Almería, Spain; ^4^Centro Médico “Avenida II”, Linares, Jaén, Spain

**Keywords:** leap motion controller, virtual reality, physiotherapy, distance learning, COVID-19, home confinement, neuroanatomy, neurorehabilitation

## Abstract

After the World Health Organization had declared a pandemic of coronavirus disease (COVID-19) on March 11, 2020 many governments, including the Government of Spain, declared the state of alarm enforcing a quarantine that have left millions of students confined to their homes. This home confinement has affected students of all levels, including university students, and has forced faculties to adapt online teaching strategies. Thus, traditional classroom face-to-face teaching has suddenly been replaced by online classes. This has revealed particularly challenging for medical courses. For such purpose we have designed an online teaching proposal addressed to the Degree in Physiotherapy and the Double Degree in Nursing and Physiotherapy of the University of Jaén (Spain). The objective is to implement an online virtual teaching protocol through the use of Virtual Reality. For such a goal, the Leap Motion Controller (LMC) will be used to teach the neuroanatomy of the brain and spinal cord and to teach and practice neurorehabilitation exercises. Along with devices like the LMC students will be asked to use Health Sciences databases in order to achieve a significative learning of the course topics. The project is structured in two phases. First, students will learn neuroanatomy and neurophysiology of the most relevant neurological conditions using LMC-based models. Then, they will learn to combine LMC games and conventional physiotherapy for neurorehabilitation purposes. The work of students will include the recording of videoreports demonstrating the acquisition of neuroanatomy concepts and simulating a clinical case. With this project we will assess the usability of LMC as an educative tool, the perception, satisfaction and self-regulated learning of physiotherapy students.

## Introduction

### Impact of COVID-19 in University Health Sciences Teaching and Learning

The World Health Organization (WHO) country office in China, reported the first case of the new coronavirus “COVID-19 (SARS-CoV-2) in Wuham (China) on December 31, 2019 (Ammar et al., 2021). Consequently, the global irruption of COVID-19 at the beginning of 2020 has caused a health alarm considered as pandemic by the WHO on March 11, 2020 (Sohrabi et al., 2020). The virus spread quickly around the world causing a large number of intensive care hospitalizations and deaths of older people (Devaux et al., 2021). With the aim to reduce the curve of contagions and deaths, health authorities of most countries established social distancing measures between population (de la Fuente et al., 2021) and declarated a home confinement and quarantine (Jiang et al., 2020) that has been the most severe disruption of normal lifestyles in history (Hossain et al., 2020).

This home confinement forced to interrupt face-to-face teaching in schools, institutes and universities (Stokes, 2020). Face-to-face teaching has been canceled in order to promote social distancing, limiting the risk that students contract the COVID-19, and become foci of infection which spread to the healthcare teaching staff who could be assigned to fight COVID-19 (Newman and Lattouf, 2020). This has caused that theoretical and practical training in healthcare courses, as well as the assessment of students’ achievements must be done at their homes (Elearning; Yang et al., 2021), adopting a new relationship between teachers and their students (Wang et al., 2020). Online teaching has been proposed as the main method for home learning (Aziz Ansari et al., 2021; Pilkington and Hanif, 2021). Although home learning is effective to reduce COVID-19 infections, it has caused devastating effects in students’ physical, emotional and social life (Cachón-Zagalaz et al., 2020; López-Castro et al., 2021). Students have showed a reduced level of physical activity during the quarantine and an increased level of psychological overload due to anxiety (García-González et al., 2021), depression (Cao et al., 2020), mood disturbance, irritability (Kostiæ et al., 2021), stress (Znazen et al., 2021), nervousness and sleep disorders (Saadeh et al., 2021), among others (Feng et al., 2021; Lavigne-Cerván et al., 2021). Related with the home confinement in education, a decreased level of attention and motivation has been reported by students during home confinement due to the difficulties to perform a normal teaching-learning process at home (Prado-Gascó et al., 2020; Zaccoletti et al., 2020). The lack of computer resources and the lack of face-to-face monitoring may be two main causes of poor academic performance during the confinement. However, to reduce students discomfort, different authors have reported the positive emotional effects of home physical exercise or active lifestyle during quarantine (Bentlage et al., 2020; Chtourou et al., 2020), which can also increase academic performance during COVID-19 (Bentlage et al., 2020).

Students of different Health Sciences, such as Medicine, Physiotherapy, Nursing or Psychology must learn multiple contents of neuroanatomy and clinical management of the most prevalent neurological diseases. Usually, these contents are learning at university laboratories and classrooms through face-to-face practical sessions in which models, corpses and role-play simulations are used. However, due to the health alarm and quarantine status the academic staff has been required to adapt their teaching taking advantage of the possibilities of tools like Virtual Reality (VR). In this sense, teachers must encourage the use of new technologies that permit students keep receiving the best possible training. Different studies have applied digital technologies to promote distance-education before COVID-19 pandemic with promising results. Besides, recent studies have showed positive psychosocial effects of the use of communication technologies during home teaching and during home physical training during home confinement (Souissi et al., 2021).

### The Importance of NeuroEducation in Physiotherapy

Anatomy learning is essential for the training of healthcare professionals, specially physiotherapist (McLachlan and Patten, 2006). Neuroanatomy defines as the science that studies the macroscopic (histology, cytology, and cell biology) and microscopic structures of the Nervous System (NS), which is composed by the Central Nervous System (CNS) and the Peripheral Nervous System (PNS) (Arantes et al., 2018). Neuroanatomy stands as one of the most difficult subjects for students (Arantes et al., 2018). One reason is the complexity of the study contents related to the CNS and PNS, which include the pathways involved in each neurophysiological process. The other reason is the shortage of anatomical tools, including neuroanatomy models, handbooks or software at universities. For these two reasons it is imperative that teachers pre-explain or provide instructions to students along with the study material (Yammine and Violato, 2016). During the quarantine period, it is essential to facilitate access to new tools that help Physiotherapy students in the learning of neuroanatomy. In addition, the emotional coping of students toward the psychological consequences of confinement can entail a loss of meaningful learning of anatomy and therapeutic contents, resulting in an academic training gap that will affect their subsequent clinical practice (Brooks et al., 2020). Social and educational isolation affecting the study (Zhai and Du, 2020) are key barriers that must be overcome through the implementation of new technologies for the study of anatomical content.

An exhaustive study of neuroanatomy and neurophysiology, as well as the most prevalent neurological disorders, their clinical manifestations and their therapeutical management is essential for the correct training of students as future physiotherapists. The success in the subsequent clinical practice is related to the training process in the university. In this sense, the COVID-19 pandemic precludes normal face-to-face teaching in the traditional classroom setting and impedes an adequate practice. Teachers must do an effort using available technologies to provide quality instruction of neuroanatomical contents during home confinement.

### New Technologies Applied to Neuroanatomy Teaching in Health Sciences: Epistemological Development

In the last two decades, a considerable increase in the use of new teaching tecnhonologies and tools has enabled the emergence of Digital Education (DE) (Martinengo et al., 2020). DE is “the fact of teaching and learning through digital technologies” (Car et al., 2019) which happens in a number of situations and scenarios such as electronic resources, offline and online education, games-based learning and virtual reality environments that allow teaching or acquisition of professional practice skills (Martinengo et al., 2019). The use of projected images, tablets, or smartphones have enabled the view of the most important neuroanatomical structures and has allowed a visual understanding of the complexity of cerebral structures (Wainman et al., 2018). However, these technologies provide 2D views and do not allow the students see neuroanatomical structures from a three-dimensional perspective, showing their size and volume. During the time of home confinement is necessary to use other digital tools that provide an immersive view and allow students interact with neurological contents, as well as a bidirectional online communication between students, their mates and teachers. That is, a virtual classroom that substitutes traditional neurology and physiotherapy teaching.

Virtual Reality (VR) technology allows teaching and interaction in a reproducible and controlled environment (Bennett and Saunders, 2019). This technology provides multisensory training and immersion (Pothier et al., 2012) in learning contents, so VR can become the perfect ally for teaching neuroanatomy during home confinement. VR increases educational possibilities in the classroom, adding a new dimension to the pre-established study program. It also enables an interactive virtual environment that makes possible students develop their own experiences and interact with learning materials (Zhao et al., 2020). Finally, and most importantly, VR ease the study of abstract contents in a graphical way.

Among a number of different VR devices, the Leap Motion Controller (LMC) (Ultraleap Ltd, 2020) has been used as a neurorehabilitation and teaching tool (Partridge et al., 2016; Sun et al., 2017). LMC is a consumer-grade, contact-free interaction and low-cost VR device designed to capture the movements of human hands and fingers (Niechwiej-Szwedo et al., 2018). It can be plugged into the USB port of a computer (Bachmann et al., 2018) and does not require to place sensors on the participant’s body (Bachmann et al., 2018). LMC allows tracking the position of the arms, wrists and hands of up to four people (Oña et al., 2018). This device includes three infrared sensors and two cameras for hand recognition (Fonk et al., 2021). LMC presents some advantages such as its small size, it is easy to use and install and can be used with different applications for physical rehabilitation (Placidi et al., 2018; Fernández-González et al., 2019; Cortés-Pérez et al., 2021; de los Reyes-Guzmán et al., 2021). Regarding the employment of LMC in teaching LMC allows the interaction of the user’s hands in a three-dimensional environment through different applications (Yang et al., 2020), including videogames. Thus, LMC is postulated as a reliable and valid tool for neuroanatomy teaching and eLearning. Different videogames allow students can select a neuroanatomical region from a virtual model and move it, flip it, or zoom in or out in the 3D space. It also permits students visualize the interior of a structure, which would be impossible in a 2D viewing. This increases the teaching realism and meaningful learning thanks to a full interaction with neuroanatomical virtual models. According to this, several authors have analyzed the use of VR and LMC in anatomy teaching and training of surgical and healthcare procedures (Ferreira dos Santos et al., 2016; Yeo et al., 2018; Kyaw et al., 2019; Alvarez-Lopez et al., 2020), or engineering training among others (Häfner et al., 2013). After using LCM, students reported that it was a useful, easy-to-use and effective device for anatomy learning, highlighting its precision for hand and finger recognition (Nainggolan et al., 2016). In addition, other forms to develop the elearning can be considered with promising results (Nieto-Escamez and Roldan-Tapia, 2021), such as gamification using VR (López Chávez et al., 2020; Vermeir et al., 2020) or without VR (Gutiérrez-Puertas et al., 2020; Molina-Torres et al., 2021).

Currently, there is scientific evidence supporting the use of VR devices in teaching and learning processes. It has been shown that it boosts students’ imagination, creativity and significative learning (Herrera et al., 2006). Various studies in neuroeducation have suggested that visual materials favor the development of brain schemas, improving long-term memory capacity and meaningful internalization of learning concepts. It has been reported that students perceive that using VR in classroom makes teaching more enjoyable and attractive, and increases their participation of Vishwanath et al. (2017). Other studies emphasize the fact that teaching through VR promotes constructivism (Alhalabi, 2016), increasing significant learning and improving analytical thinking thanks to cognitive modifications associated to the use of more complex tools (Passig et al., 2016). A recent meta-analysis reinforces the idea that didactic interventions with VR produce a positive effect on the expected results and increases students’ significant knowledge. Moreover, compared with 2D digital methods, VR can improve the effectiveness of teaching in anatomy curses (Zhao et al., 2020). Therefore, VR and especially the LMC is postulated as a useful and plausible tool for studying Health Sciences courses at home, such as neuroanatomy in the degree of Physiotherapy. In addition, it has been reported that 90% of students agree with the use of 3D visualization techniques applied to neuroanatomy courses and believe that they provide a more effective learning (Nainggolan et al., 2016).

### Emotion and Self-Regulated Learning, Cognitive, and Emotional Variables

The majority of studies in educational areas have mainly focused on the cognitive processes of students, leaving the emotional processes in a second level (Frenzel et al., 2018). Perhaps, this is because teaching has traditionally focused on students’ cognitive and behavioral development to assimilate a large amount of information (Barajas and Gannaway, 2007). However, emotions associated with the events experienced by the students can make easier to remember what has been studied whereas learnings remain for longer in their memory. The use of experiences that students feel as favorable and respond to their interests will facilitate the development of positive emotions (for example, fun, pride, and confidence), leading to a facilitation of the memory and knowledge linked to such events (Pell et al., 2009). Conversely, negative emotions (e.g., boredom, anxiety, frustration) interfere with and may even inhibit the recall, as it makes difficult to store information into memory and retrieve it during the task performance, impairing learning (Di Gesú and Seminara, 2012). If we also consider distance teaching, it has been reported that it requires high levels of motivation (Morris et al., 2005).

Additionally, people exposed to social isolation and quarantine have an unpleasant experience (Brooks et al., 2020), so the current situation due to COVID-19 may also have negative psychological consequences (Brooks et al., 2020; Wang et al., 2020). The motivation of students may be affected with the lack of contact with classmates, teachers and professionals in training centers, leading to a deterioration of their interest and satisfaction levels (Balaban-Sali, 2008). For instance, it has been reported a direct relationship between the quality of online teaching and students satisfaction (Palmer and Holt, 2009). It is therefore necessary to assess student satisfaction as a measure of the quality of remote learning.

It is also necessary to bear in mind that remote learning entails a component of self-regulated learning, which is made up of three components: metacognitive strategies (planning, supervision and regulation of one’s own cognition), control and management of one’s own effort, and cognitive strategies (referring to cognition itself; Pintrich and De Groot, 1990). This project will therefore pay special attention to resources control and management strategies, as well as to metacognitive strategies by students in home learning situation. For that reason, it is necessary to study the learning process itself, particularly the type of strategies that students develop in self-regulated learning.

### Justification of This Project

Healthcare professionals working with patients suffering from neurological disorders, especially physiotherapists, need to know the structure and anatomy of the Central and Peripheral Nervous Systems; as well as the most prevalent and physically disabling neurological diseases, along with conventional therapies for their treatment. In recent years, the advances in the field of neurorehabilitation have led to the introduction of VR devices for the treatment of these patients. In this sense, knowing coadjuvant techniques for rehabilitation together with the traditional procedures becomes essential. Physiotherapy students must continue their training during the period of quarantine and teachers must find new solutions to promote distance teaching and meaningful learning at home. Moreover, in order to generalize and establish the psychoeducational foundations for the use of VR tools in online Physiotherapy learning it is essential to know and assess how it affects students’ satisfaction and their motivational, cognitive, metacognitive, resources control and management strategies.

## Pedagogical Framework

This e-learning proposal has been designed for the course *“Specific Methods of Intervention in Physiotherapy III”* in which the LMC device will be employed for the study of neuroanatomy and the main clinical manifestations of prevalent neurological disorders (stroke, multiple sclerosis and Parkinson diseases among others) susceptible of receiving Physiotherapy treatment. LMC will help students develop a realistic online training and will promote significative learning of neuroanatomy and neurorehabilitation. This project will be implemented as a pilot in one group of students who will receive a LMC and the results will be compared with an equivalent group of students enrolled in the same course who will receive more traditional online learning.

### Methodology

The proposed project will start with an initial introduction about LMC. In these pre-initial instructions about LMC, teachers will do a search in Health Science databases (Medline PubMed, Scopus, Web of Science or PsyCINFO ProQuest, among others) to show students how LMC can be used in neuroanatomy learning and neurorehabilitation training. The University of Jaen offers the possibility of using digital platforms for bidirectional online communication between students and teachers. Students will have access to documentation and one LMC at home for the entire duration of the course. A work schedule similar to the official teaching guide will be available to them.

Learning contents included in this course focus on the study of different neurological disorders, and their neuroanatomical correlates and main clinical symptoms. In this order, students will receive training about stroke, acquired brain injury, multiple sclerosis, spinal cord lesions, and neurodegenerative disorders such as Alzheimer or Parkinson disease. Each pathology will be developed as a learning unit. The learning model for each learning unit will be organized in periods of 2 weeks according to the following schedule:

1. **Common Pre-initial Phase**: Before teaching starts, every student will receive one LMC device by postal service. Students must sign a device cession deal with the University of Jaen. At the same time, teachers will send an e-mail to students with the instructions about the use of the LMC device. In this phase, students will learn the initial use of LMC, how to install and update it, and the required software. Teachers will provide an access code for differents 3D anatomy softwares and VR neurorehabilitation games to be used in each course unit.
2. **Neuroanatomical and Neuropathology Teaching Phase (Week 1)**: For every learning unit, teachers will provide online theoretical contents about the selected pathology and its neuroanatomy during the first week. *Google’s Gsuite Meet* platform will be used for online classes. Students work will consist in reviewing theoretical contents about neuroanatomy and neurophysiology using LMC. Teachers will show the brain and/or spinal cord regions through different 3D virtual anatomical models with LMC. It will allow students visualize and interact with the learning contents. LMC allows different images of the brain and the spinal cord with a great number of projections. After the online face-to-face lecture, students must use LMC for neuroanatomy learning, performing a number of virtual practices. At the end of the week, students will record a video demonstrating the learning of the proposed neuroanatomy contents using LMC. This recording must be delivered the first day of the second week. In addition, students will do an online exam where they must identify, in real time, using LMC any neuroanatomical structure studied in the current learning unit.
3. **Virtual Neurorehabilitation Teaching and Practicing Phase (Week 2):** In this phase students will learn to design a neurorehabilitation protocol for the pathology studied in phase 1. Students will be asked to combine conventional Physiotherapy (CP) and LMC. Initially, teachers will provide information about the main CP techniques for the management of neurological diseases. A study document will be sent to the students along with references where these techniques are used. This document will contain concepts about the benefits of passive and active mobilizations; specific methods like Proprioceptive Neuromuscular Facilitation (FNP), Bobath, Le Metayer, Brunnstrom; Voja and counsels to prevent complications. Then, the LMC will be presented as a therapeutic tool for clinical settings or home rehabilitation. Thus, students will be instructed to use LMC combined with CP. Theoretical contents about the use and benefits of VR in neurorrehabilitation will be explained to students. Teachers will show differents VR games to be used with LMC in clinical practice. Finally, students must carry out a simulated clinical case for the selected neurological pathology by using CP and LMC games. This simulation must be recorded in video showing how to use LMC and CP for patient’s neurorehabilitation. A family member or a neurological patient living with them can participate in the simulation of the neurorehabilitation protocol. During this phase teachers must provide continuous feedback to students regarding their treatment proposal. The recorded simulation showing the proposed treatment will be used by teachers to evaluate the performance of students.

During the whole process of remote learning teachers will provide continuous feedback to students. Students will be also asked about the difficulties found during the learning process. Any feedback about technical problems and their solutions will be essential to improve teaching in further units. Table 1 illustrates the course planning as well as the evaluation schedule.

**TABLE 1:** Project stages.

		Week	
		
Phases	Scheduled tasks	1	2
1	Reception of theoretical content about neurological pathology and involved neuroanatomical area	X	
	*G-Suite Meet* face-to-face teaching sessions with LMC	X	
	Practical activities to learning neuroanatomy using LMC	X	
	Recorded video using LMC to learn neuroanatomy	X	
	Individualized online practical exam using LMC	X	
2	Teacher sends a document about the use of CP and LMC in neurorehabilitation of the neurological disease revised in the didactic unit		X
	Neurorehabilitation training with CP techniques		X
	Students are taught about the use of LMC in neurorehabilitation		X
	Development and recording of a neurorehabilitation protocol combining CP and LMC		X

*Phase 1, Neuroanatomical and neuropathology teaching phase; 2, Virtual neurorehabilitation teaching and practicing phase; CP, Conventional physiotherapy; LMC, Leap motion controller.*

At the end of the teaching-learning process of every learning unit, students will be individually evaluated about theoretical and practical learnings.

### Objectives

This project is conceived to assess the feasibility of a remote learning course during COVID-19 home confinement addressed to the study of neuroanatomy and neuropathology by physiotherapy students. Students must: (1) be able to integrate theory and practice contents by using virtual anatomical models; (2) design an appropriate Physiotherapy neurorehabilitation protocol combining conventional procedures with VR tools using LMC; (3) get used with new VR-based tools, particularly LMC, for neurorehabilitation of sensorio-motor sequels in neurological diseases; (4) demonstrate how to use LMC as a neurorehabilitation tool based on active movement; (5) assess the acceptance of LMC-based virtual learning of neuroanatomy through the System Usability Scale; Finally (6) we will assess the effect of remote learning through LMC in self-regulated learning variables.

### Required Resources

**FIGURE 1:**
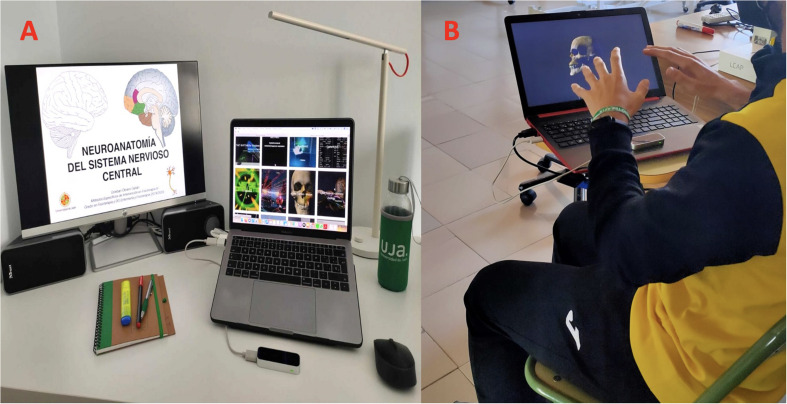
**(A)** Typical students’ workplace including the leap motion controller. **(B)** Student using leap motion controller.

Figure 1 illustrates the necessary materials to carry out this project, including hardware, software and Internet connection. Regarding hardware, an LMC device is required. This device allows students to handle and interact with neuroanatomical images in order to carry out a significative learning. This device requires a laptop or desktop computer. After a previous survey it has been found that all students enrolled in the course have a computer and internet connection, so they will be able to work at home with this device. Currently we only have 15 LMC devices available, thanks to the collaboration with the University of Almería, which have been randomly sent to students. For the coming year we will require the University of Jaén to purchase 70 LMC devices under the premise that literature has shown that VR and LMC improve significative learning of Health Sciences concepts. To record the practice with LMC, the University of Jaen provides free access to Google’s Gsuite Meet video platform.

Regarding software needs for Neuroanatomy and Neuropathology learning, we will use *Online Anatomical Human*, an open platform compatible with LMC that provides 2D and 3D anatomical information for educational purposes (Smit et al., 2016). According to Nainggolan et al. (2016), 3D neuroanatomy figures can be created using BodyParts3D, the Database Center for Life Science, Japan^1^. In addition, other apps related to neuroanatomy, such as *Cyber Science-Motion*, a free gaming software from Leap Motion App Store (Cyber Science Motion, 2020) may be used in this project as secondary software.

For the Virtual Neurorehabilitation Teaching and Practicing Phase we will choose games designed for LMC. Active video games are a task-oriented intervention which positively influence movement rehabilitation through increased practice of motor skills, and considered an interesting alternative treatment (Wuang et al., 2011; de Oliveira et al., 2016). The objectives of rehabilitation with LMC are to improve the joint range of motion, muscle strength, coordination, and fine motor functions of the hand and wrist in patients (Tarakci et al., 2019).

The games that the students will use in phase 2 are:

1. Free and accessible games for desktop PC from Leap Motion Gallery (Ultraleap Ltd, 2020):
  a. *Sculpting*: The aim of this LMC game is to sculpt different figures and forms using the hands. Patients with upper limb and hand disorders can use this game to improve its manual function.
  b. *Takt-Rhythm*: This LMC game goal is to play music in different spaces. Patients must touch different musical notes that appear in the screen, developing their manual skills and coordination.
  c. *V2 Playground*: In this LMC game users can grab and move robots in a virtual dance club. In addition, patients can stroke flower petals. This game, employed in a neurorehabilitation process, may allow patients develop their hand and fingers fine motor skills. In addition, the whole upper limb movement is necessary.
  d. *Joca-The Handglider*: In this game, the user must to control a plane with the movement of the hand and the elbow. The plane has to be driven in different spaces solving obstacles. This game is really interesting for a neurorehabilitation protocol due to development of the manual dexterity in ludic task.
  e. *Sortee:* This LMC game consist in selecting and throwing rubbish in different bins. Objects appear quickly and patients must to use their hands and fingers to sort it out. This game works the cognitive component along with manual dexterity.
  f. *Spider Escape 3D*: This LMC game requires a precise control of wrist, hand and fingers. Patients must use their upper limb to impulse a spider in order to obtain points. This game can be used in patients with less upper limb disorders, as it requires some precision.
  g. *PopPop!* The patient has to shoot a gun with a simple pinch of the fingers. It requires a high level of precision. This game is a good option to rehabilitate thumb and index finger movement.
  h. *Froggle:* In this LMC game users must guide a frog across lily pads solving obstacles and collecting positive scores. This game is useful to work coordination and manual dexterity with a cognitive component.
2. Other games used in movement rehabilitation studies are:
  a. *Leapball:* is designed to focus on the development of grasping and single motor skills of the hand, to improve fingers dexterity and coordination, to improve hand flexion and extension abilities, to increase wrist and fingers joint range of motion, to improve movement speed, muscle strength, and motor control. In “Leapball” game, the user must grasp a virtual ball with all the fingers and to throw it into a bucket of the same color. The size of the ball can be reduced to provide progression to the ball (Tarakci et al., 2019).
  b. *CatchAPet*: is aimed to hit the rabbits coming out of the holes with repetitive wrist flexion/extension movements. Rabbits come out randomly from the holes. The player sees the avatar of his/her hand on the screen and hits the rabbits coming out of the hole by doing consecutive wrist flexion/extension movements. The faster the rabbits are hit, more points are earned (Tarakci et al., 2019).

### Feasibility and Viability of the Project

**FIGURE 2:**
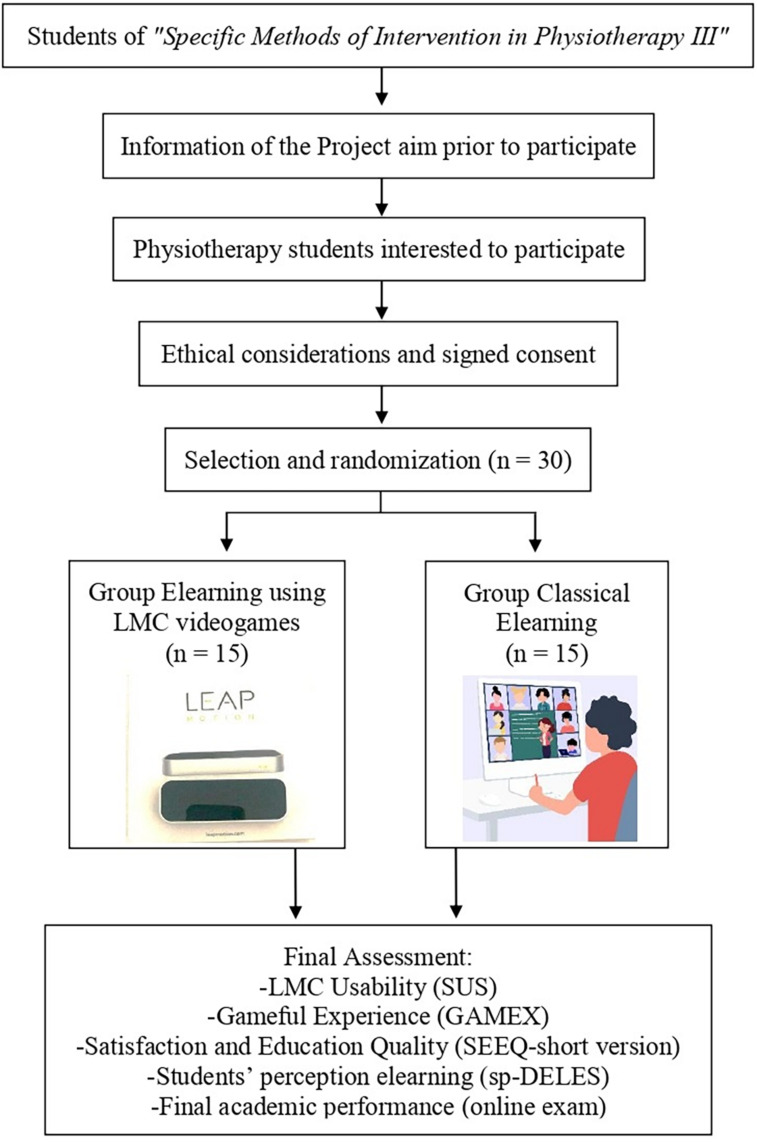
Flow diagram.

Around 75 students enroll every year in the course *”Specific Methods of Intervention in Physiotherapy III.”* From those students interested in participating voluntarily in this project, 30 students will be randomly selected and divided into two groups. Fifteen students will be allocated to the LMC-based learning group and 15 to the classical online learning group. This initial project will serve as a pilot of a larger study that will take place when a larger number of LMCs are available (Figure 2). Two teachers will be in charge of the online teaching process and student’s supervision, and will maintain a fluid communication with students through the use of G-Suite Meet tools, so they do not feel isolated during the learning process. Clinicians will also participate in every unit showing their practical experiences with patients and therapies. Materials requirements are more limited since at this time there are not LMC for not every student. The assignment of the devices should not extend longer than the course duration. We believe that this project is viable both in potential cases of future home confinement episodes or just to implement new online possibilities for neuroanatomy and neurology teaching.

## Indicators of Students Progress and Assessment of Self-Regulated Learning Variables

Teachers will assess the student’s progress across every study unit through online theory and practice exams, and the assessment of two videos recorded by students. Regarding videoreports, one of them will demonstrate the use of LMC for neuroanatomy learning. The second videoreport will illustrate how to develop a neurorehabilitation protocol combining CP and LMC in a clinical case simulation. Regarding the two online test, a practical exam using LMC to identify neuroanatomical structures will be carried out. The other exam will assess theoretical contents corresponding to every study unit. A weighted average mark will be calculated from all study unit score. Table 2 shows the rubric with the proposed scoring for each phase activities.

**TABLE 2:** Evaluation rubric for theorical and practical contents.

Neuroanatomy recorded video using LMC (Maximun 2 points)	Neurorehabilitation recorded video using LMC (Maximun 3 points)	Individualized and practical test online using LMC (Maximun 1 point)	Individualized theoretical online test of the end of didactid unit (Maximun 4 points)
Student makes the recording fluently describing the main anatomical regions proposed in the theoretical content: **1 point**	Student show a complete knowledge about the studied pathology, necessary to the correct functional and neurological Physiotherapy’s diagnos: **0.5 points**	Answer correctly 50% of the questions: **0.5 points**	Answer correctly 50% of the questions: **2 points**
Student is able to expand the didactic content by locating structures not corresponding to said thematic unit: **1 point**	Students identifies correctly the main motor disorders in the proposed clinical case: **1 point**	Answer correctly 75% of the questions: **0.75 points**	Answer correctly 75% of the questions: **3 points**
	Student establishes an appropriate protocol using CP techniques and LMC: **1 point**	Answer correctly all questions: **1 point**	Answer correctly all questions: **4 points**
	The LMC games proposed are adequate to the characteristics of the patient: **0.5 points**		

*The delay in the delivery of the recorded videos will suppose a penalty of 10% proportional in the qualification. It is necessary to answer correctly 50% of online test questions to pass this didadic unit.*

First, the usability or management of the LMC as teaching device will be assessed. The Spanish Version of the System Usability Scale (sv-SUS) for the assessment of Electronic Tools will be used. The sv-SUS is a tool used to assess the usability of a system, and was initially developed in English (Brooke, 1996). It is a self-administered questionnaire of 10 Likert-type questions with 5 response options, from 1 (strongly disagree) to 5 (strongly agree) and higher scores mean a greater usability of the system (Brooke, 2013). The sv-SUS is easy to understand, has shown a high internal reliability (Cronbach α-value of 0.812), and a face validity index of 0.94 (Sevilla-Gonzalez et al., 2020).

In this project, different LMC videogames will be used. So, the experience of physiotherapy students using these videogames will be assessed with the Spanish version of the Gameful Experience (GAMEX) (Parra-González and Segura-Robles, 2019). GAMEX is Likert scale ranging from 1 (never) to 5 (always) for 27 items distributed into six different domains: enjoyment, absorption, creative thinking, activation, absence of negative effects and dominance (Eppmann et al., 2018). This scale has shown a high reliability with a Cronbach α-value of 0.855. The Cronbach α for different dimensions are 0.843 for enjoyment, 0.898 for absorption, 0.865 for creative thinking, 0.790 for activation, 0.841 for absence of negative effects and 0.860 for dominance (Márquez-Hernández et al., 2019).

Students’ satisfaction as a measure of teaching quality will be studied with the Spanish version of Student Evaluation of Educational Quality (SEEQ)-Short version questionnaire (Bol Arreba et al., 2013). This is a five points Likert-type scale with 10 items that analyzes: Students’ interest in the subject, the use of teaching materials, teachers’ interest in the subject, teachers’ accessibility during tutoring time and outside class hours, the usefulness of teaching materials, the continuous assessment process and its usefulness, the adjustment of the evaluation procedures, the quality and clarity of teaching materials, teachers’ capability of motivation for increasing students’ participation in the class and the workload of the subject (Marsh, 1987). The Spanish version of SEEQ-Short version has shown a high reliability with a Cronbach’s α coefficient of 0.92 and significant correlations among all its domains (Bol Arreba et al., 2013).

The student’s perception of the online learning environment will be analyzed as a measure of teaching quality at the end of the project by using the Spanish version of the Distance Education Learning Environments Survey (sp-DELES) (Fernández-Pascual et al., 2015). This instrument includes seven scales: instructor support, student interaction and collaboration, personal relevance, authentic learning, active learning, autonomy and student‘s satisfaction (Walker and Fraser, 2005). The sp-DELES has shown a good reliability (Cronbach’s α coefficient between 0.86 and 0.97) and the original structure of six factors has been replicated and accounts for 72.9% of the total variance (Fernández-Pascual et al., 2015).

Besides, self-regulated learning strategies in participant students will be evaluated with the CEVEAPEU questionnaire (Gargallo et al., 2009). It is an 88-item five points Likert-type scale developed for assessing learning strategies in university students consisting of two scales. The first scale is related to Affective, Support and Control Strategies and includes subscales about Motivational Strategies, Affective Components, Metacognitive Strategies, Control of Context, Social Interaction and Resources Management Strategies. The second part of CEVEAPEU assesses Information Processing Strategies. It includes two subscales related to Information Search and Selection Strategies, and Strategies of Information Processing and Use. The CEVEAPEU questionnaire has shown a high reliability (Cronbach’s α coefficient of 0.897; Gargallo et al., 2009).

Finally, students’ academic performance will be evaluated in both groups through an test with 30 items.

For the analysis and treatment of data, the statistical package IBM SPSS Version 20.0 will be used (SPSS, 2020).

## Expected Results

We pretend to remark the importance of introducing virtual learning strategies to get a successful training during the home confinement period, when students with low motivation or bad study habits may fail in the learning process. We consider that the use of VR tools will reduce such disadvantages, and students involved in the project will develop significative learning of the topics studied, as well as practical and professional abilities. Thus, it is feasible that students included in this methodology will obtain better marks than students following traditional or E-Learning methodologies as result of an increase of their motivation and development of self-regulated learning strategies. Moreover, students will also be able to identify new potential uses for VR tools in neurorehabilitation, as well as to propose new physiotherapy treatments that combine classic neurorehabilitation and VR therapy.

A previous work by Stephan et al. (2017) reported that students were highly motivated and showed higher participation and understanding of lectures through the use of virtual environments compared to blended learning methodology. Nevertheless, they informed that students reported some difficulties using VR tools. We expect these difficulties will be overcome by initial training on the use of LMC and the initial feedback received from students that will be used to improve subsequent activities. A recent work by Harfouche and Nakhle (2020) have shown that VR can be implemented in distance learning as a useful tool to motivate students, encouraging interactive learning, developing critical thinking and decision-making skills. Nevertheless, to our knowledge, there are not studies that assess how VR solutions affect specific learning strategies, and potentially improve those strategies necessary for a successful self-regulated learning. We will assess how the introduction of VR affects these variables through a validated questionnaire (CEVEAPEU).

By the end of this project it is expected an increase of students’ motivational strategies, particularly intrinsic motivation, higher task value and self-efficacy perception. These variables are particularly relevant for self-regulated learning (López Paz et al., 2018) and will be linked with an improvement of information search and selection strategies as students must actively search information in databases and apply it to the course tasks. We expect that it will positively affect information processing and use, including information acquisition, development, and planning, critical thinking and memorization. Metacognitive strategies are also expected to improve, with an enhancement of control and self-regulation, linked to knowledge of learning goals and evaluation criteria which are explicit from the beginning of the course. This will positively affect learning planification and self-assessment. Finally, it is expected an increase of information transference and use of learnings as students can find a high applicability of virtual simulations in professional contexts.

In self-regulated learning strategies also must consider emotional variables. We expect an improvement of affective components, with a reduction of anxiety that would be associated to the perception of social support in the learning process during confinement by teachers and other students.

Regarding the opinion of students about teaching quality, we expect positive results in the assessment with the SEEQ-short form and sp-DELES questionnaire. In this sense, the continuous assessment process and feedback proposed by this project will result in an improvement of teaching quality during home confinement.

When home confinement period ends this project can still be used in our course, reinforcing face-to-face classroom work at home. It would be ideal that students can loan LMC devices to be used in other courses of Physiotherapy studies. It will require we also train our colleagues. With this goal, the promotors of this project will offer informative sessions and seminaries about the use of LCM and the other VR devices susceptible to be used as a teaching tool. We expect our results encourage teachers of Physiotherapy Studies to use virtual teaching in the future, both in case of a new confinement episode or just as a e-learning valuable tool.

## Project Constraints

To promote the use of LMC during home confinement and later as useful virtual teaching tool, we must overcome logistics and financial barriers. In this sense, LMC has a competitive price and it just requires a computer, specific anatomical 3D software and Internet connection. Not all students have the same possibilities of accessing technology or Internet connection, so universities should make an effort to provide them the necessary resources to study at home during confinement and get advantage of LCM attributes for neuroanatomy teaching. As an initial measure, the University of Jaen provides support to low income students free Internet connection. If the home confinement were extended over time during the beginning of the next academic year, the University would have all the necessary materials so new students will not have difficulties to participate in the e-learning project.

## Conclusion

LMC postulates as an inexpensive VR device useful in the teaching-learning process of neuroanatomy and neurorehabilitation topics for Health Sciences studies, particularly in Physiotherapy degrees. Besides, VR technology has already shown satisfactory results and higher levels of interaction than 2D and 3D images and videos. The combination of VR and digital imagery together with the analysis of clinical cases will become a good learning strategy that will increase students’ motivation and adherence to virtual e-learning during home confinement. However, according with recent studies, it will be also essential to promote an active lifestyle during home leaning aimed to reduce the negative emotional consequences of social distancing and increase mental health to improve academic performance during home confinement. In addition, teachers’ commitment will play a fundamental role to accompany the student in the acquisition of the objectives proposed in the project. This project shows the flexibility of Physiotherapy teachers at the University of Jaén to adapt to the changes motivated by COVID-19.

## Data Availability Statement

The original contributions presented in the study are included in the article/Supplementary Material, further inquiries can be directed to the corresponding author/s.

## Author Contributions

EO-G, FN-E, NZ-A, and IC-P conceived and developed this project. EO-G, NZ-A, and IC-P wrote the manuscript. FN-E revised and verified the final version. All authors have read and accepted the content of the manuscript.

## Conflict of Interest

The authors declare that the research was conducted in the absence of any commercial or financial relationships that could be construed as a potential conflict of interest.

## Footnotes

1 http://doi.org/10.18908/lsdba.nbdc00837-000
